# New Appliances and Things Medical

**Published:** 1904-01-16

**Authors:** 


### NEW APPLIANCES AND THINGS MEDICAL.

### ALLENBURY'S MILK FOOD CHOCOLATE.

(MESSRS. ALLEN AND HANBURY, LIMITED, BETHNAL GREEN, LONDON, E.)

THE milk food manufactured by the above firm is well known to be scientifically adapted to the use of hand-fed infants. This preparation consists of 25 per cent. of this milk food combined with pure chocolate, thus constituting a most nutritious food in a digestible and concentrated form, and possessing a highly delicious flavour. The large percentage of fat contained renders preparations of this kind, if judiciously administered, most valuable to young children who are unable to take cod-liver oil or other forms of fat. Adults will also find this Milk Chocolate useful when travelling or when requiring sustenance in small bulk.

### PEPTONISED INVALID BISCUITS.

(NURSE WEBB, 25 CHORLTON GROVE, CHORLTON ROAD, BROOKS'S BAR, MANCHESTER.)

THESE biscuits are made principally from rice flour with a small amount of cornflour, enriched by the addition of butter and eggs, and sweetened with saccharine, the whole being peptonised before baking. They are placed on the market with a view to partially replacing in an invalid's dietary some of the liquid peptonised dishes with which patients are continually deluged. These biscuits, being predigested, palatable, and carefully prepared, should prove a useful adjunct to other foods.

### STUDENT'S SET OF QUANTITATIVE ANALYSIS.

(REBMAN PUBLISHING COMPANY, LIMITED, 129 SHAFTESBURY AVENUE, LONDON, W.C.)

MR. BARKER SMITH has devised a rapid method for the estimation of alcohol, urea, ammonia, albuminoids, etc., by thermometry. In estimating the percentage of alcohol present in a liquid, the process consists in mixing a certain volume of the liquid at a known temperature with an equal quantity of water at the same temperature. After briskly shaking up the mixture, the resultant rise of temperature is in ratio to the volume of alcohol present, the amount of which is ascertained by reference to a table. In the case of urea, ammonia, and albuminoids, hypochlorite of sodium solution is used instead of water, and the rise of temperature multiplied by a certain factor gives the amount of these constituents in the sample of urine. The presence of glucose in urine can also be estimated in a somewhat similar way. Such methods which can be carried out in a short space of time and entail the use of such simple apparatus, should, if found to be sufficiently accurate for clinical purposes, prove extremely handy to both medical men and students, and tend to promote the desirable habit of routine urine analysis.

